# Transvenous Embolization of Olfactory Groove Dural Arteriovenous Fistula: A Case Series of Three Patients

**DOI:** 10.7759/cureus.106957

**Published:** 2026-04-13

**Authors:** Amir Nazari, Michihiro Tanaka, Kotaro Ueda, Takafumi Mitsutake, Keisuke Kadooka

**Affiliations:** 1 Department of Neurological Surgery, International School of Medicine, Istanbul Medipol University, Istanbul, TUR; 2 Department of Neuroendovascular Surgery, Kameda Medical Center, Kamogawa, JPN

**Keywords:** anterior cranial fossa, dural arteriovenous fistula, endovascular treatment, olfactory groove, transvenous embolization

## Abstract

Olfactory groove or anterior cranial fossa (ACF) dural arteriovenous fistulas (DAVFs) are rare but highly aggressive lesions because of their direct cortical venous drainage and high risk of intracranial hemorrhage. Transarterial embolization (TAE) is commonly considered the first-line treatment; however, anatomical complexity or arterial limitations may make TAE technically difficult or unsafe.

We present a case series of three patients with olfactory groove DAVFs successfully treated using transvenous embolization (TVE). In all cases, the fistulous point was accessed through the superior sagittal sinus and cortical bridging veins, allowing targeted coil embolization of the draining vein. Complete angiographic obliteration was achieved in all patients.

One patient developed neurological deficits related to a thromboembolic event during arterial catheterization, while the remaining two patients had uneventful recoveries.

Our experience suggests that TVE can be a feasible and effective treatment option for selected olfactory groove DAVFs, particularly in cases where arterial access is challenging or when previous transarterial treatment has failed.

## Introduction

Anterior cranial fossa (ACF) dural arteriovenous fistulas (DAVFs), particularly those located at the olfactory groove and crista galli, represent a rare subset of intracranial DAVFs but are considered among the most aggressive because of their direct cortical venous drainage and high risk of intracranial hemorrhage [1]. Early diagnosis and treatment are therefore recommended once the lesion is identified.

Traditionally, treatment options have included microsurgical disconnection and transarterial embolization (TAE). TAE is often feasible because the anterior ethmoidal artery arising from the ophthalmic artery frequently serves as the main arterial feeder. However, TAE may be limited by complex arterial anatomy or the potential risk of central retinal artery compromise [2,3].

In such situations, transvenous embolization (TVE) or mixed TVE/TAE may represent an alternative treatment strategy. By targeting the draining vein near the fistulous point, TVE can achieve complete occlusion while avoiding the manipulation of the ophthalmic arterial circulation [4-7].

Although a few reports have described the feasibility of venous approaches in ACF DAVFs, published series remain limited [4]. In this study, we present three patients with olfactory groove DAVFs successfully treated using a purely TVE strategy.

## Case presentation

Case 1 

A 59-year-old male patient presented with dizziness and vertigo. Magnetic resonance imaging (MRI) demonstrated abnormal venous dilation on the left frontal lobe. Cerebral angiography revealed an arteriovenous shunt at the level of the cribriform plate supplied by the anterior ethmoidal artery arising from the ophthalmic artery.

Venous drainage occurred through a frontal cortical vein entering the anterior third of the superior sagittal sinus (SSS).

Under general anesthesia, 6-Fr guiding catheters were introduced through the right femoral artery, and a 6-Fr guiding sheath was introduced through the right femoral vein.

Bilateral external carotid angiography (ECAG) did not reveal any shunts; however, left internal carotid angiography (ICAG) demonstrated a small arteriovenous shunt at the level of the crista galli (olfactory groove). The feeding artery was identified as the anterior ethmoidal artery arising from the ophthalmic artery. To obtain a superselective image of the lesion, a microcatheter was navigated to the left ophthalmic artery. Subsequently, thromboembolic occlusion of the left M1 segment of the middle cerebral artery (MCA) was observed.

Mechanical thrombectomy using both a stent retriever and an aspiration catheter was immediately performed, resulting in successful recanalization. 

**Figure 1:**
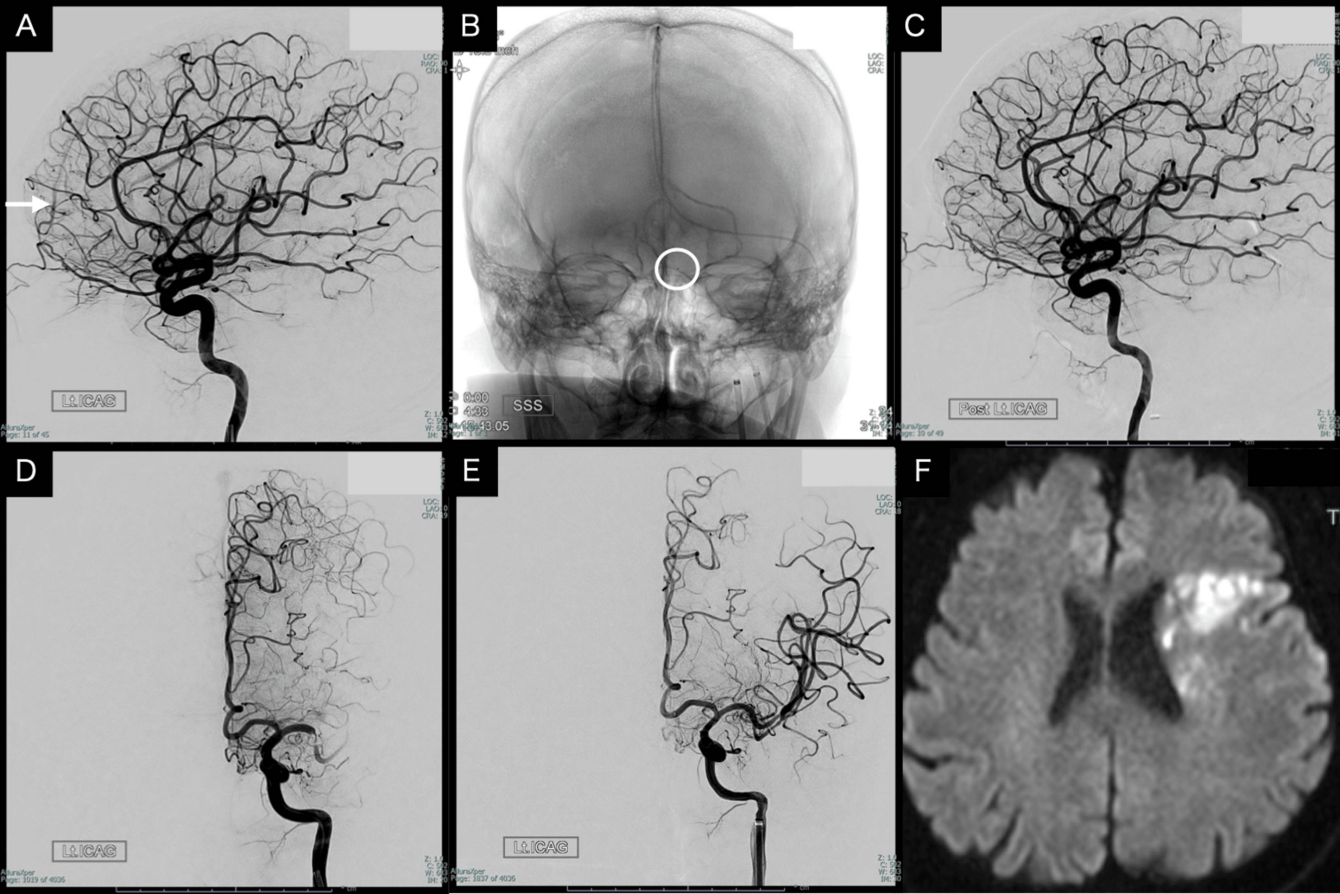
TVE of the first patient (A) Preoperative left ICAG. The white arrow indicates the draining vein. (B) Microcatheter positioned at the foot of the draining vein near the shunt point (centered within the white circle). (C) Postoperative left ICAG demonstrating the complete obliteration of the shunt. (D) Intraoperative occlusion of the left M1 segment. (E) Recanalization of the same vessel following mechanical thrombectomy. (F) Postoperative MRI (DWI) demonstrating an infarct in the left frontal lobe. TVE: transvenous embolization; ICAG: internal carotid angiography; MRI: magnetic resonance imaging; DWI: diffusion-weighted imaging

A TVE was performed afterwards through the SSS and the frontal cortical vein using a distal access catheter (Guidepost 3.2/3.4 Fr, 130 cm, Tokai Medical Products, Aichi, Japan) along with a microcatheter (Marathon, Medtronic, Dublin, Ireland). The tip of the catheter was successfully placed at the foot of the vein of the shunt point, and 28 cm of coil in total were used in the bridging vein of the left anterior olfactory groove. Control angiography of the bilateral external and internal carotid arteries showed complete elimination of the DAVF with the preservation of the central retinal artery and ophthalmic circulation (Figure 1). 

Postoperatively, the patient developed right hemiparesis and motor aphasia, which gradually improved during rehabilitation. 

At the six-month follow-up, the patient was independent in activities of daily living with mild residual motor aphasia. At the one-year follow-up, the aphasia had resolved, and the patient achieved a modified Rankin Scale (mRS) score of 0.

There was no evidence of recurrence during two years of MRI follow-up.

Case 2

A 66-year-old male patient underwent MRI screening for an unruptured aneurysm. During diagnostic angiography, an olfactory groove DAVF was incidentally detected. 

Four months after aneurysm treatment, embolization of the DAVF was performed under general anesthesia. Right ICAG demonstrated a shunt supplied by the right anterior ethmoidal artery from the ophthalmic artery. Venous drainage occurred through a frontal cortical vein entering the anterior SSS. The shunt point was located at the olfactory groove. Left ICAG also showed a small shunt at the same location as the right side. The draining vein through the bridging vein of the right side of the crista galli and the bilateral ECAG showed no marked contribution to the shunt.

A microcatheter (1.5-Fr Baltacci, Balt, Montmorency, France) along with a Chikai 008 microwire were navigated into the orifice of the right ophthalmic artery for clear shunt visualization, and subsequently, with the use of a 4-Fr distal access catheter (Cerulean G, Medikit, Tokyo, Japan), the TVE was performed through the jugular vein and eventually the anterior segment of SSS. Afterwards, the microcatheter (Komichi 155 cm, HI-LEX, Takarazuka, Japan) was successfully guided to the foot of the vein in the right cribriform plate level. 

Using a total of 17 cm coils in the bridging vein of the anterior olfactory groove, complete elimination of this DAVF was achieved; however, a small amount of extravasation was observed which we occluded using additional coils.

Control angiography confirmed the elimination of the DAVF while preserving the central retinal artery and ophthalmic system. Stagnation was observed in the cortical artery of the right posterior parietal branch and posterior temporal artery, however, which was resolved after the heparin administration.

**Figure 2:**
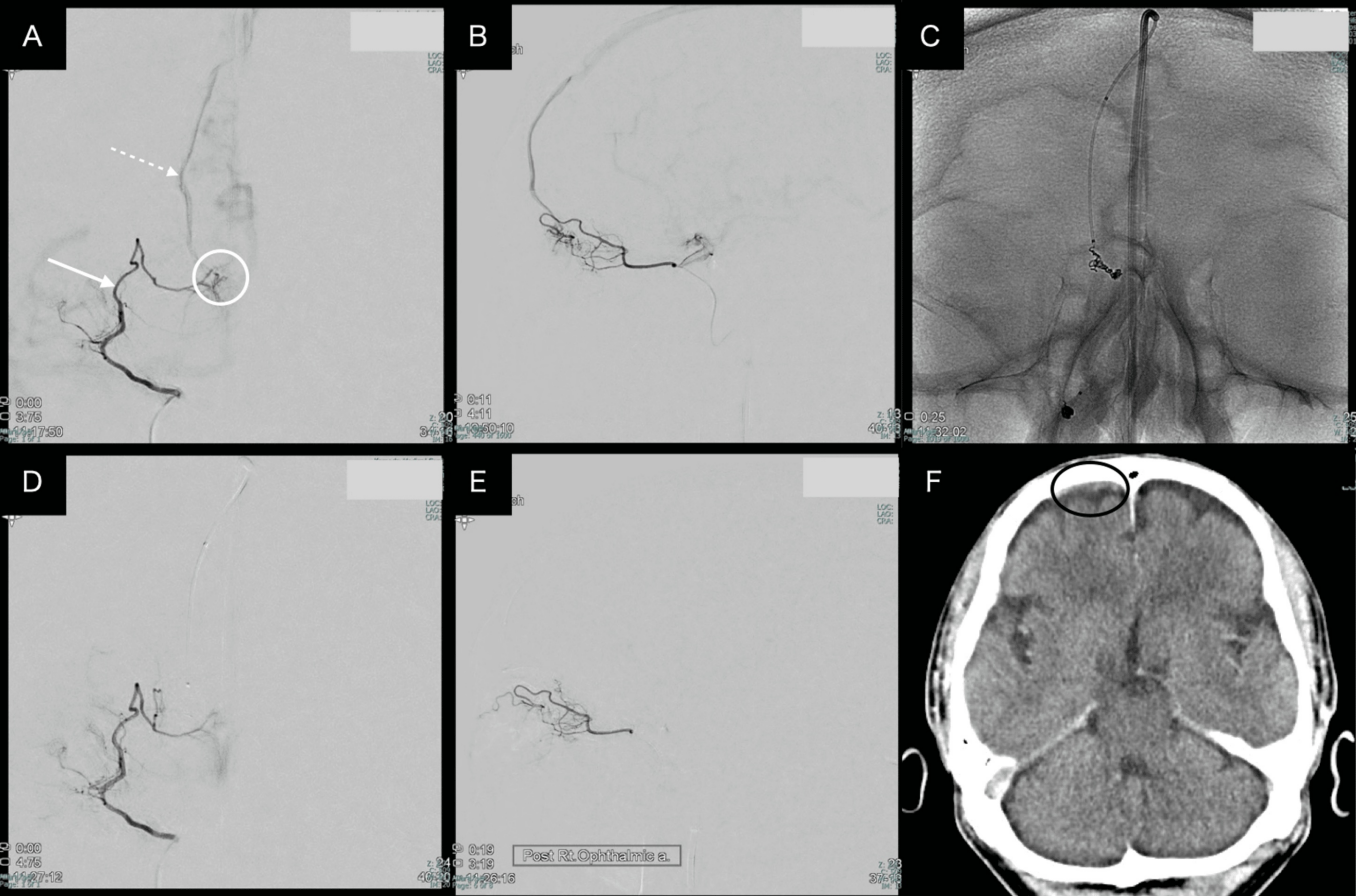
TVE of the second patient (A) Preoperative right ophthalmic artery angiography (white arrow) demonstrating the anterior ethmoidal artery supplying the shunt point (centered within the white circle), with a draining vein (white dashed line) visualized over the surface of the right frontal lobe. (B) Lateral view of right ophthalmic artery angiography. (C) Plain radiograph during TVE showing the microcatheter positioned at the shunt point with coil deployment in progress. (D) Postoperative anteroposterior view of right ophthalmic artery angiography demonstrating the complete obliteration of the shunt. (E) Postoperative lateral view of right ophthalmic artery angiography confirming the complete obliteration of the shunt. (F) Postoperative non-contrast CT demonstrating a small amount of hemorrhage over the surface of the right frontal lobe. TVE: transvenous embolization; CT: computed tomography

The patient emerged from anesthesia without any sequential symptoms and deficits and was later discharged home on postoperative day 3 with an mRS score of 0 (Figure 2).

No recurrence was observed during four years of MRI follow-up.

Case 3 

A 54-year-old male patient with a left olfactory groove DAVF had previously undergone TAE. However, complete occlusion was not achieved because the glue did not reach the shunt point, resulting in the proximal occlusion of the anterior ethmoidal artery. 

Subsequently, the fistula recurred with recruitment of arterial supply from the ethmoidal branch of the sphenopalatine artery via the external carotid artery.

Under general anesthesia, 6-Fr guiding catheters were introduced through the femoral artery and vein. Bilateral ICAG did not show any arteriovenous shunts, while the ECAG showed a small shunt at the level of the olfactory groove. 

The terminal feeding artery was determined to be the ethmoidal branch of the sphenopalatine artery, originating from the maxillary artery. 

A distal access catheter (4-Fr Cerulean G) combined with a microcatheter (Marathon) was navigated through the right jugular vein, passing the transverse sinus. The distal access reached the anterior part of the SSS. Subsequently, Marathon was navigated and placed in the initial foot of the vein at the level of the left cribriform plate, and platinum coils were deployed.

**Figure 3:**
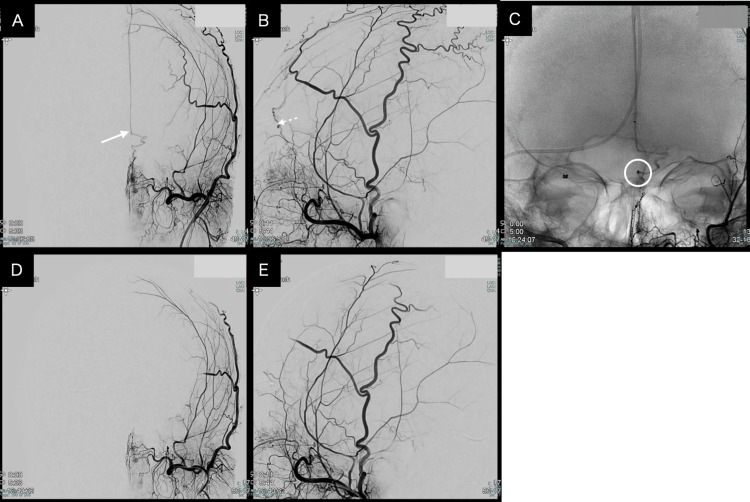
TVE of the third patient (A) Preoperative anteroposterior view of left external carotid artery angiography demonstrating a DAVF supplied by the anterior ethmoidal artery arising from distal branches of the sphenopalatine artery. The white arrow indicates the point where the draining vein enters the superior sagittal sinus. (B) Preoperative lateral view of left external carotid artery angiography. The white dashed line indicates the draining vein. (C) Left external carotid artery angiography during TVE demonstrating the microcatheter advanced into the draining vein with coil deployment in progress. (D) Postoperative anteroposterior view of left external carotid artery angiography demonstrating the complete obliteration of the shunt. (E) Postoperative lateral view of left external carotid artery angiography confirming the complete obliteration of the shunt. TVE: transvenous embolization; DAVF: dural arteriovenous fistula

Control angiography demonstrated the complete obliteration of DAVF, and the patient was discharged home on postoperative day 3 with an mRS score of 0 (Figure 3).

No recurrence was observed during four years of MRI follow-up.

The clinical characteristics of the three patients are shown in Table 1.

**Table 1:** Summary of the presented cases DAVF: dural arteriovenous fistula; TAE: transarterial embolization; SSS: superior sagittal sinus; TVE: transvenous embolization

Case	Age/sex	Symptoms	Terminal feeder	Venous drainage	Prior therapy	TVE approach and devices	Embolic material	Outcome
1	59 M	Dizziness, vertigo	Anterior ethmoidal artery from ophthalmic artery	SSS via frontal cortical vein	None	Right jugular vein → SSS → draining vein; distal access catheter + microcatheter	Coil	Complete occlusion: hemiparesis and motor aphasia (recovered)
2	66 M	Incidental finding during aneurysm treatment	Anterior ethmoidal artery (bilateral contribution)	SSS via frontal cortical vein	Aneurysm embolization (prior, unrelated)	Right jugular → SSS → bridging vein; microcatheter + distal access catheter	Coil	Complete occlusion; minor extravasation, no neurological sequelae
3	54 M	Recurrent DAVF after TAE	Anterior ethmoidal artery from sphenopalatine artery	SSS via frontal cortical vein	TAE	Right jugular → SSS → draining vein; microcatheter + distal access catheter	Coil	Complete occlusion; no complications

## Discussion

Olfactory groove/ACF DAVFs, although rare, are among the most aggressive types of DAVFs, primarily because they are non-sinus-type lesions with direct shunt flow into the cortical venous system. In addition, the presence of cortical venous reflux and a high tendency for hemorrhage further contribute to their malignant clinical behavior [1]. Most reported cases have been treated with either microsurgical disconnection or a TAE approach, given the relatively accessible route through the anterior ethmoidal artery arising from the ophthalmic artery [2,3]. Although TAE is generally effective, it carries inherent risks, including central retinal artery occlusion [2,3].

TVE has gradually emerged as an alternative treatment strategy in selected cases. Previous reports have demonstrated that coil embolization via a venous approach can achieve the complete obliteration of the fistula [4]. More recently, combined TAE and TVE approaches have been advocated to improve safety and efficacy [5-7]. A recent multicenter study further demonstrated that TVE achieved a significantly higher angiographic cure rate compared to transarterial approaches in ACF DAVFs, supporting the efficacy of this strategy [8]. All three cases in our series were successfully treated using a purely transvenous strategy, preserving the ophthalmic arterial circulation. 

In our cases, venous drainage occurred through frontal cortical veins entering the anterior third of the SSS. This venous configuration provided a relatively short and straight access route, facilitating safe microcatheter navigation from the SSS into the draining vein and enabling effective coil embolization near the fistulous point. This anatomical feature may partly explain the technical feasibility of TVE in these patients.

A thorough understanding of the venous anatomy is essential for successful treatment. Careful navigation through the SSS and cortical bridging veins towards the cribriform plate is critical to avoid venous injury. Dense coil packing at the foot of the draining vein allows the durable occlusion of the shunt while minimizing the risk of recurrence. In our series, no recurrence was observed during follow-up periods of two, four, and four years, respectively, supporting the durability of this approach. Although no specific flow-control techniques such as arterial wedging or induced hypotension were routinely employed in our cases, careful coil deployment at the shunt point is necessary to minimize the risk of hemorrhagic complications.

In one case, a thromboembolic event occurred during arterial catheterization while evaluating the angioarchitecture of the lesion. Although this complication was successfully treated with mechanical thrombectomy, it highlights the potential risks associated with arterial manipulation in these lesions. In another case, minor contrast extravasation occurred during coil deployment but was immediately controlled with additional coils and did not result in clinical sequelae.

Although TAE is generally considered the first-line treatment, our experience suggests that TVE may be particularly advantageous in cases with unfavorable arterial access or in recurrent lesions. In addition to the risk of reflux into the ophthalmic circulation, TAE may also carry a risk of inadvertent embolization into the anterior cerebral artery territory via the olfactory artery. In this regard, a transvenous approach may offer an additional safety advantage by avoiding arterial manipulation in this region.

The limitations of this study include the small sample size and its retrospective nature. Nevertheless, our findings support the safety and efficacy of TVE for olfactory groove DAVFs, either as a primary treatment or as an alternative when TAE is not feasible. Larger multicenter studies are warranted to further define the optimal indications for this approach.

## Conclusions

TVE can be an effective treatment strategy for selected olfactory groove DAVFs. In this series, complete angiographic occlusion was achieved in all patients through the coil embolization of the draining vein. This approach may be particularly useful when arterial access is difficult or when previous transarterial treatment has failed.

## References

[1] Matsubara S, Takai H, Enomoto N (2022). Anterior cranial fossa osseous arteriovenous fistula of the crista galli with bone erosion: patient series. J Neurosurg Case Lessons.

[2] Zhang G, Pang M, Duan G (2025). Transarterial embolization of anterior cranial fossa dural arteriovenous fistulas as a first-line approach: a retrospective single-center study. Acta Neurochir (Wien).

[3] Voldřich R, Charvát F, Netuka D (2023). Copolymer liquid embolization of dural arteriovenous fistulas: a 20-year single-center experience. J Neuroimaging.

[4] Defreyne L, Vanlangenhove P, Vandekerckhove T, Deschrijver I, Sieben G, Klaes R, Kunnen M (2000). Transvenous embolization of a dural arteriovenous fistula of the anterior cranial fossa: preliminary results. AJNR Am J Neuroradiol.

[5] Sugihara M, Fujita A, Ikeuchi Y (2023). Combined transarterial and transvenous embolization of anterior cranial fossa dural arteriovenous fistula. Surg Neurol Int.

[6] Maciejewski K, Pinkiewicz M, Mruk B, Knap D, Zaczyński A, Walecki J, Zawadzki M (2025). A practical approach to intracranial dural arteriovenous fistulas: pathogenesis, classification and management. J Clin Med.

[7] Trivelato FP, Smajda S, Saleme S (2022). Endovascular treatment of anterior cranial base dural arteriovenous fistulas as a first-line approach: a multicenter study. J Neurosurg.

[8] Dabus G, Kan P, Diaz C (2021). Endovascular treatment of anterior cranial fossa dural arteriovenous fistula: a multicenter series. Neuroradiology.

